# Modeling of surface phenomena of liquid Al–Ni alloys using molecular dynamics

**DOI:** 10.1038/s41598-023-31844-w

**Published:** 2023-03-21

**Authors:** Hadassa Juárez, Ensieh Yousefi, Anil Kunwar, Youqing Sun, Muxing Guo, Nele Moelans, David Seveno

**Affiliations:** 1 Department of Materials Engineering, KU Leuven, Kasteelpark Arenberg 44, Box 2450, 3001 Leuven, Belgium; 2 Faculty of Mechanical Engineering, Silesian University of Technology, Konarskiego 18A, 44-100 Gliwice, Poland

**Keywords:** Materials science, Nanoscale materials, Nanoparticles, Nanoscale materials, Structural properties

## Abstract

This work presents a study on the surface tension of liquid Aluminum–Nickel (Al–Ni) alloys. Obtaining adequate values of surface tension for this system is not a simple task as these alloys present the formation of atomic clusters with short-range order at certain compositions, which dramatically influences surface tension. The Compound Forming Model predicts the influence of these clusters on surface tension, but experimental limitations have obstructed its validation due to deficient thermodynamic data. This work attempts to overcome some of these limitations by using Molecular Dynamics (MD). By comparing the obtained results from MD simulations with those of an equivalent system without clusters, it was possible to infer the role of the atomic clusters on Al–Ni surface tension. It was found that these clusters increase surface tension by decreasing the Al content at the surface. They achieve this reduction in Al content at the surface by trapping Al atoms and hindering their travel to the surface.

## Introduction

Due to their good corrosion resistance and thermal stability, Aluminum–Nickel (Al–Ni) alloys are considered a good choice for structural applications at high temperatures^1, 2^. An additional advantage is their relatively low weight, with up to 15% reductions compared with other similarly used alloys, such as Nickel–Chromium (Ni–Cr). Production and manufacturing of these alloys require extensive and reliable knowledge of their properties, including surface tension which plays a significant role in the castability of the melt. Additionally, this property can affect the resulting solidification structure and the presence of defects. During the welding procedure, surface tension affects the heat distribution and weld penetration dynamics and thus has a significant role in the reliability of the joints^3^. These showcase the importance of studying the surface tension of liquid Al–Ni alloys. Many experimental attempts have been made to obtain the surface tension of pure Al^4, 5^, Ni^6^, and Al–Ni alloys^1, 7–10^. Historically, the relationship between surface tension and composition of these alloys has always been a topic of continued interest since Al–Ni alloys show behavior different from that of most binary alloys. The general relationship between surface tension and the composition of a liquid A–B binary alloy has been modeled by Butler (Eq. 1)^11^. Butler provides an extension to the model by considering the surface of a liquid as an additional thermodynamic phase in equilibrium with the bulk^11^.

1 $$\gamma = \gamma_{i} + \frac{{N_{A} k_{B} T}}{\alpha }\ln \left[ {\frac{{c_{i}^{s} }}{{c_{i}^{b} }}} \right] + \frac{{N_{A} k_{B} T}}{\alpha }\ln \left[ {\frac{{a_{i}^{s} }}{{a_{i}^{b} }}} \right]$$

where $\gamma$ is the surface tension of a liquid binary alloy, $\gamma_{i}$ is the surface tension of the pure component *i*, $N_{A}$ is the Avogadro’s number, $k_{B}$ is the Boltzmann constant, $T$ is the temperature (K), $c_{i}$ is the concentration of the component *i*, $a_{i}$ is the activity of the component *i*. The superscripts $s$ and $b$ are used to indicate quantities referred to the surface and the bulk, respectively. The subscript $i$ is used to denote the corresponding alloy component, and can adopt the values $i = A,{ }B$^12^. $\alpha$ is the mean molar surface area of the alloy (For more details about obtaining $\alpha$ see the supplementary material).

The relationship between surface tension and composition greatly depends on the degree of interaction between atoms A and B, which is different for ideal, regular, and real solutions. For an ideal solution, it is hypothesized that there is no difference between A–A, A–B, and B–B pair interactions^13^. In this case, Eq. (1) reduces to:

2 $$\gamma = \gamma_{i} + \frac{{2N_{A} k_{B} T}}{\alpha }\ln \left[ {\frac{{c_{i}^{s} }}{{c_{i}^{b} }}} \right]$$

Another approach to obtain the surface tension of an ideal alloy was proposed by Guggenheim^14^:

3 $$Exp\left[ {\frac{ - \gamma \alpha }{{k_{B} T}}} \right] = \mathop \sum \limits_{i} c_{i}^{b} \left[ {\frac{{ - \gamma_{i} \alpha }}{{k_{B} T}}} \right]$$

with the same meaning of $\gamma$, $\gamma_{i}$, $\alpha$, $c_{i}^{b}$, $T$, and $k_{B}$ as explained above. For more details about the model suggested by Guggenheim, see supplementary material.

Bernard and Lupis^15^ used the Sessile Drop method to measure the surface tension of the Gold–Silver (Au–Ag) binary system. Their results showed that the Au–Ag system could be described as an ideal solution. The main difference between ideal and regular solutions is that the interactions between A–A, A–B, and B–B are no longer considered to be equal. Nonetheless, the interactions between dissimilar (i.e., A–B) atoms are considered weak, and thus a random configuration is energetically most favorable^13, 16^. The Quasi-Chemical Approximation (QCA) for a regular solution can be used to model the relationship between surface tension and composition^17^. Based on this model, the relationship between surface tension and composition of regular alloys is described as:

4 $$\gamma = \gamma_{i} + \frac{{N_{A} k_{B} T}}{\alpha }\ln \left[ {\frac{{c_{i}^{s} }}{{c_{i}^{b} }}} \right] + \frac{{N_{A} k_{B} T}}{\alpha }\left( {p\ln \left[ {\frac{{a_{i}^{s} }}{{a_{i}^{b} }}} \right] - q ln\left[ {a_{i}^{b} } \right]} \right)$$

where $p$ and $q$ are surface coordination fractions, see the Quasi-Chemical Approximation section in the supplementary material for more details about this model.

Plevachuk et al.^18^ measured surface tension of Bismuth–Tin (Bi–Sn) alloys at 550 K, using the Sessile Drop method. There was a good agreement between their results and the QCA model. For ideal and regular solutions, the surface tension is expected to decrease monotonously by increasing the component's content with the lowest surface tension. Therefore, the composition versus surface tension curve presents a characteristic concave shape.

Real solutions are different from regular solutions as the interaction between dissimilar atoms becomes stronger. Due to this, a random configuration is no longer the most energetically favorable one, and the atoms will tend to form groups^13^. The Compound Forming Model for strong interactions (CFM) detailed in^19^ and^20^ is then relevant when the tendency is to form A–B groups. Its main characteristic is that it includes the presence of *A*_η_*B*_υ_ complexes (η and υ are the number of A and B atoms), or clusters, in the melt. Evidence of these short medium range order structures has been found using X-Ray diffraction^7, 8, 21^. For example, Brillo et al.^21^ studied the local short-to-intermediate range structure in liquid Al–Ni and Aluminum–Copper (Al–Cu) alloys. Apart from the regular peaks in the structure factor curves, a distinct pre-peak was observed, which indicated the presence of Al–Ni and Al–Cu clusters at certain compositions. Donatella et al.^22^ used the large drop method to measure surface tension and density of Al–Ni alloys as a function of composition and temperature. They observed that the behavior of Ni-rich alloys could be well-described by the CFM model. Das et al.^23^, who also performed neutron scattering tests and Molecular Dynamics (MD) studies on Al–Ni melts at 1795 K, confirmed a presence of pre-peaks in the structure factor, indicating the formation of Al–Ni clusters. It can be concluded that the Al–Ni system shows behavior that deviates largely from the ideal solution. The presence of these clusters could completely modify the surface tension of Al–Ni alloys. In the same work, Das et al.^23^ studied the influence of clusters on Al–Ni surface tension by comparing their results to the QCA and CFM. Their findings suggested that the presence of clusters increases surface tension by decreasing the surface Al content. This study, however, assumes a priori that (1) the CFM is indeed an appropriate model for Al–Ni, (2) the data from thermodynamic databases are adequate, and (3) the effect of the high reactivity of Al–Ni system is negligible. These assumptions may be questionable, particularly because CFM does not provide surface tension predictions with good accuracy. Additionally, the accuracy and validity of thermodynamic databases for these uses has been questioned before^24^. Finally, even though experimental data was obtained using the Oscillating Drop technique, the high reactivity of Al–Ni can lead to surface contamination such as by oxygen and vaporization^25^. The limitations mentioned are accompanied by natural restrictions in the experimental setup. Due to these restrictions, to the best of the author’s knowledge, there are no published studies that provide precise information about, for example, the formation, size, or lifetime of clusters, as well as their influence on surface tension. To overcome these limitations, a simulation method, like Molecular Dynamics (MD), has great potential for predicting physical–chemical properties and understanding complex phenomena. MD has been used extensively to predict the surface tension of pure^26–28^ and binary metals^29, 30^. For example, Kunwar et al.^29^ predicted surface tension of pure Al and interfacial energies for Al, Ni, and Al_3_Ni_2_ using MD. Calvo^30^ analyzed the surface tension of Ag–Au alloys by MD with a tight binding force field. The behavior was close to that of an ideal solution; the surface tension decreases as the content of the element with the lowest surface tension increases. Despite having the same trend as found experimentally, there was a difference of up to 30% between experimental and MD values. Shin et al.^31^ studied Aluminum–Iron–Nickel (Al–Fe–Ni) binary alloys using a ReaxFF force field. This study predicted the surface composition of a single alloy. Their findings showed the segregation of Al atoms at the surface, i.e., higher Al content at the surface than the bulk, which is in agreement with the CFM model. Their study does not include any surface tension measurement. As in every other MD study, enough care should be taken to select a reliable force field. As Webb and Grey proposed^32^, a charge gradient correction coupled with the appropriate EAM potential can provide surface tension values that are more accurate. However, this does not exclude the existence of EAM force fields capable of accurately predicting surface events in the absence of charge gradient correction.

In this current study, MD was used, together with the Butler, Guggenheim, QCA, and CFM, to understand how the formation of Al–Ni atomic clusters with short-range order modifies the surface tension of liquid Al–Ni alloys. Different systems of Al–xNi (x is a ratio of Ni content in the system) at 2000 K were investigated in terms of surface composition determination and cluster behavior, such as their formation and dissociation. The Embedded Atom Method (EAM) potential developed by Zhou et al.^33, 34^ is used to describe the multi-particle interactions due to the advantages and improvements with respect to other EAM potentials.

## Materials and methods

### Force field

Interactions between Al and Ni can be modeled by a few force fields, namely, EAM developed by Baskes et al.^35^ and Mishin et al.^36–38^, EAM + charge transfer ionic (CTI) developed by Zhou et al.^33, 34^, ReaxFF^31, 39^, and COMB^40^. The choice of a force field was first based on its reliability in predicting the surface tension of pure Al. It was then calculated using these 7 force fields and compared to experimental results (see Force field section in the supplementary material). The most accurate one was EAM + CTI introduced by Zhou, which was then selected to study the interactions between Al and Ni. Down to a concentration of 30% in Al, this force field also predicted surface tensions of the alloys, which are in agreement with experimental observations as shown hereafter. In our previous publication^26^, we showed that this force field is also the most accurate one in predicting the self-diffusion coefficient.

### Simulation setup

Firstly, it is desirable that the obtained results are independent of the model characteristics. In this work, the effect of initial configuration (see Figs. A.1 and A.2 in the supplementary material) and system size (see A.3 in the supplementary material) were tested to evaluate their influence on the surface tension of 50 at% Al-50 at% Ni system. It was concluded that the findings of this work were mostly independent of the prescribed simulation parameters (see the simulation setup in the supplementary material). As a result, the following procedure was used to simulate Al–Ni systems. Firstly, an FCC slab made of 50 400 Al atoms with dimensions 80.5 × 80.5 × 124.0 Å^3^ and lattice spacing of 4.05 Å was constructed. Secondly, Al atoms were randomly substituted with Ni atoms until the desired composition of an alloy was reached. The studied compositions were Al (pure Al), Al–0.1Ni (10 at% Al–90 at% Ni), Al–0.2Ni (80 at% Al–20 at% Ni), Al–0.3Ni (70 at% Al–30 at% Ni), Al–0.4Ni (60 at% Al–40 at% Ni), Al–0.5Ni (50 at% Al–50 at% Ni), Al–0.6Ni (40 at% Al–60 at% Ni), and Al–0.7Ni (30 at% Al–70 at% Ni). Notice that the total number of atoms (sum of Al and Ni atoms) was the same for all compositions. Thirdly, an FCC crystal was heated up using NVT ensemble (constant number of elements, volume, and temperature) and Nose/Hoover thermostat from 300 to 2000 K at a heating rate of 0.01 K fs^−1^^41^.

**Figure 1:**
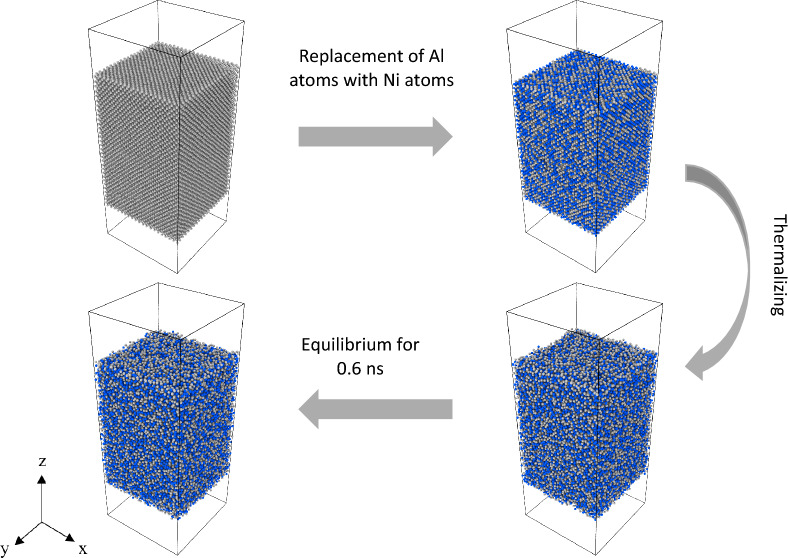
Al–0.5Ni system preparation. Aluminum and Nickel atoms are colored gray and blue respectively.

The dimension of the simulation box in the z-direction was adjusted so that the Al–Ni structures could expand freely when melting. Periodic boundary conditions were applied to the simulation box in all directions. Finally, after 0.6 ns when the systems were at an equilibrium state (Fig. A.4 in the supplementary material), data were collected to measure the target properties. Figure 1 shows the preparation procedure for the Al–0.5Ni system. The Velocity-Verlet algorithm^42^ was applied to solve Newton’s equation of motion at each time step of 1 fs. The simulations were performed using the LAMMPS software developed by Sandia National Laboratories^43^. The Open Visualization Tool (OVITO)^44^ was used To visualize the simulations.

### Properties measurement

#### Composition profile

The system was divided into slices of 1 Å in the $z$ direction, and the number of Al and Ni atoms was used to obtain the Al content at each slice. The properties were calculated by averaging them over the last 0.4 ns.

#### Surface identification

In an alloy-vapor system, the density ($\rho$) smoothly transitions from the bulk value of the alloy, ρ_*l*_, to the bulk value of the vapor, ρ_*v*_. Based on^45^, the position and thickness of the surface were determined by fitting the density profile to Eq. (5).

5 $$\rho \left( z \right) = \frac{1}{2}\left( {\rho_{l} + \rho_{v} } \right) - \frac{1}{2}\left( {\rho_{l} - \rho_{v} } \right)\tanh \left[ {2\frac{{z - z_{0} }}{\omega }} \right]$$

where *z*_0_ is the position of the surface and ω is the surface thickness. For this study, ρ_*v*_ = 0 as the phase in contact with the alloy is the vacuum. Therefore, the alloy-vapor interface was found at the position where $\rho = \rho_{l} /2$.

#### Surface tension

According to Irving and Kirkwood, surface tension $\left( \gamma \right)$ can be calculated by slicing the system and adding the difference between normal and tangential components of the pressure tensor at each slice^46^. This method is commonly known as “the mechanical approach” and employs Eqs. (6)–(8).

6 $$\gamma = \frac{1}{2}\mathop \smallint \limits_{ - \infty }^{ + \infty } \left( {P_{N} \left( z \right) - P_{\tau } \left( z \right)} \right)dz$$

7 $$P_{N} \left( z \right) = P_{zz} \left( z \right)$$

8 $$P_{\tau } \left( z \right) = \frac{1}{2}P_{xx} \left( z \right) + \frac{1}{2}P_{yy} \left( z \right)$$

where *P*_*N*_(*z*) and *P*_τ_(*z*) are the normal and tangential components of the pressure tensor, respectively. Pressure includes the kinetic term and virial term. The kinetic term originates from the kinetic energy, whereas the virial term originates from the pairwise forces between atoms. The virial term was computed as described by Thompson et al.^47^. *dz* is the slice thickness, which in this work corresponds to 0.2 Å. We collected data when stable surface tensions were obtained (see Fig. A.5 in the supplementary material).

#### Ideal and non-ideal solutions

Obtaining chemical potentials or similar properties from MD is quite a laborious process. If the system presents strong interactions, as in Al–Ni, it may not be possible to calculate them [56]. As a consequence, the Butler model was used to obtain an activity ratio, defined as:

9 $$a_{Al}^{R} = \frac{{a_{Al}^{s} }}{{a_{Al}^{b} }}$$

$a_{Al}^{R}$ could be measured using Eq. (1):

10 $$a_{Al}^{R} = e^{{\frac{{\alpha \gamma - \alpha \gamma_{Al} - k_{B} T ln\left[ {\frac{{c_{Al}^{s} }}{{c_{Al}^{b} }}} \right] }}{{k_{B} T}}}}$$

Besides, the compositions at the surface and the bulk are used to define a concentration ratio according to Eq. (11).

11 $$c_{Al}^{R} = \frac{{c_{Al}^{s} }}{{c_{Al}^{b} }}$$

The Al activity and concentration ratios,$a_{Al}^{R}$ and $c_{Al}^{R}$, were used to infer whether a specific alloy behaved as an ideal or non-ideal solution. An alloy was said to behave similarly to an ideal solution when its $a_{Al}^{R}$ was equal to the corresponding $c_{Al}^{R}$, and non-ideal otherwise^13^.

#### Cluster identification

**Figure 2:**
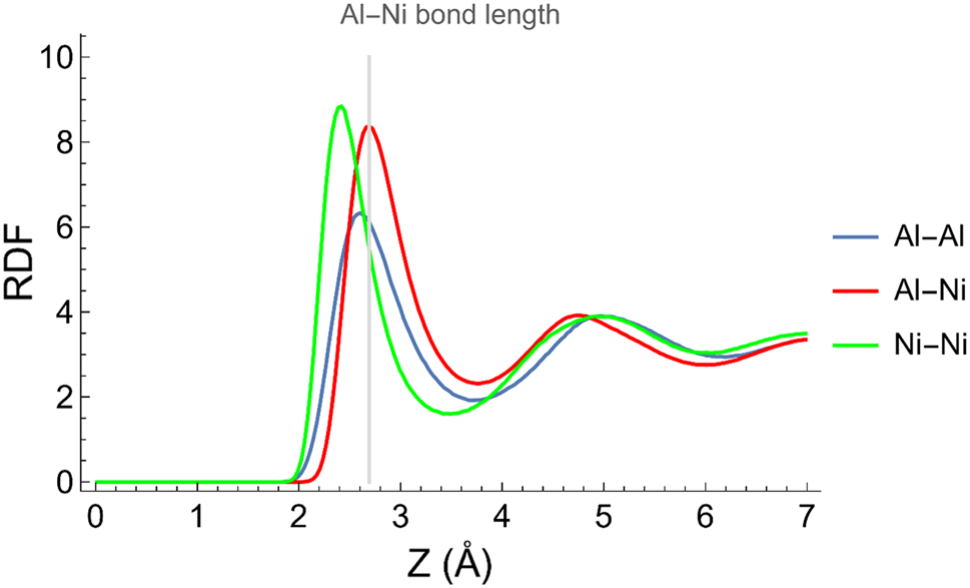
Radial distribution function of elements in Al–0.5Ni and bond length of Al–Ni (For interpretation of the references to color in this figure legend, the reader is referred to the web version of this article).

The methodology used to determine the presence of Al–Ni clusters in each alloy was divided into several steps. Firstly, the radial distribution function (RDF) was computed. Then, the Al–Ni bond length was determined from the first peak position of the partial Al–Ni RDF (Fig. 2). In this study, the Al–Ni bond length was determined to be 2.68 Å, consistent with experimental and numerical studies^33, 48–50^. Next, the pairwise distance between all the atoms was calculated, and the pairs with a distance between them corresponding to the Al–Ni bond length were selected. Finally, groups or clusters were identified by picking which of these selected pairs had atoms in common.

## Results and discussion

### Surface tension

**Figure 3:**
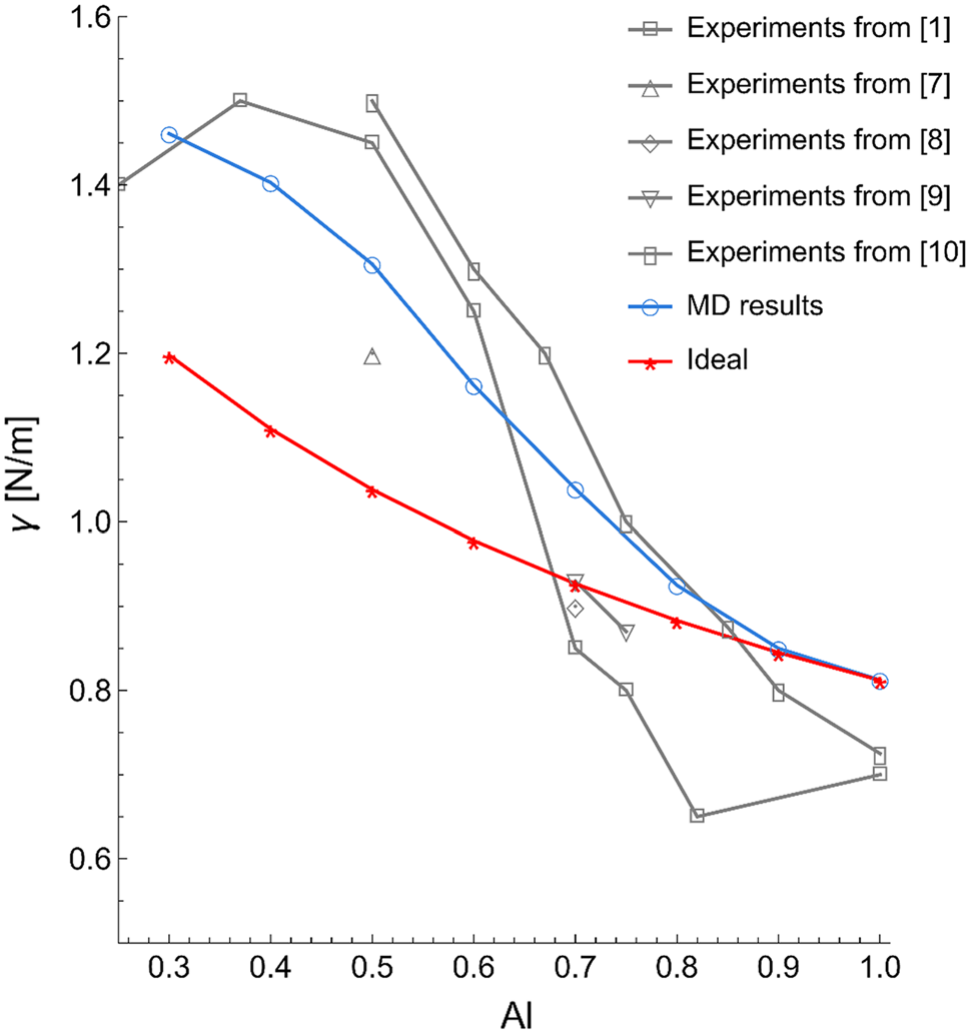
Comparison of surface tensions of Al–Ni alloys obtained using MD, the ideal solution model, and experiments (surface tension error bars were not presented since they were less than 0.05% of the absolute values).

Figure 3 shows the surface tension of Al–Ni systems obtained by MD (blue curve) and equivalent ideal one (red curve) using the Guggenheim equation (Eq. 3). Surface tension obtained by MD was measured by averaging over 400,000 steps (0.4 ns) when the system reached almost equilibrium. Therefore, the fluctuations of pressure in this condition were minor. For instance, the surface tension value for the Al–0.5Ni system was 1.30640 ± 0004 N m^−1^ when the error bar was derived using standard deviation. Error bars were less than 0.05% of the absolute values. Two crucial conditions must be satisfied. Firstly, we should obtain surface tension values comparable with experimental results. Second, the behavior of a real solution should be mimicked since Al–Ni alloys are not ideal or regular solutions. Figure 3 compares MD results (blue curve) with other experimental data (gray curves). The surface tension values are within the experimental range^1, 7–10^, illustrating the high accuracy of the MD results. Not only surface tension of Al–Ni alloys but also surface tension and surface energy of pure Al and Ni using this force field are within experimental range. In our previous publications^26, 29^, we showed that this force field is accurate in prediction of surface tension and surface energy of pure Al and Ni at different temperatures. The predictions obtained from MD are also compared to the ideal and regular behavior (red curve). The latter shows a concave shape, while the MD results show a characteristic non-concave shape, typical of a real solution. These two observations indicate that we can study the surface tension behavior of Al–Ni systems using MD. Data from the MD simulations should provide novel insight into the surface phenomena of these alloys. This is addressed in more detail in the following sections.

### Surface identification and composition

By fitting the density profile with Eq. (5), *z*_0_ and ω were obtained for each alloy. The Al content at the surface was then determined using these two quantities with the corresponding composition profile.

**Figure 4:**
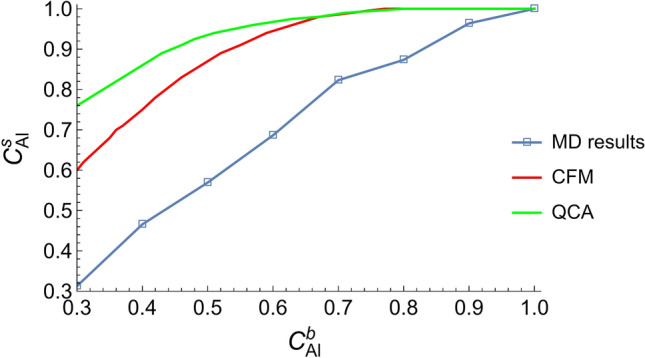
Surface composition of Al–Ni alloys as obtained using MD and the regular (QCA) and real (CFM) model (For interpretation of the references to color in this figure legend, the reader is referred to the web version of this article).

As seen from Table 1, all alloys featured Al surface segregation, i.e., higher Al content on the surface than in bulk. As there is no available experimental data for the surface composition of liquid Al–Ni alloys, it is reasonable that the comparison between MD and experiments can be replaced by the comparison between MD and the CFM or QCA model. Care should be taken, though, because the surface determined by these thermodynamic models may not accurately reflect the actual surface. While, in contrast, in MD study, the surface composition of Al–Ni alloys was literally directly measured. For these reasons, it was considered best to compare the general trends between $c_{Al}^{s}$ obtained from MD and $c_{Al}^{s}$ from CFM/QCA rather than the numerical values. Surface composition predicted by CFM and QCA were extracted from^25^. Figure 4 shows that the general trend of surface segregation at all compositions obtained from MD matches the one predicted by CFM and QCA. However, higher Al segregation at the surface of the alloys is predicted by both thermodynamic models (CFM/QCA), especially at higher Ni content. As mentioned before, the accuracy and validity of these models are under question due to the inadequate thermodynamic databases. Additionally, CFM does not provide spectacular surface tension predictions, which are inferred from the results of^25^. On the other hand, MD results depend on the force field, and care should be taken when interpreting simulation results. These deficiencies can explain the difference between MD results and the thermodynamic models in Fig. 4.

**Table 1:** Surface composition (Al content at the surface) of Al–Ni alloys.

System	${\mathrm{c}}_{\mathrm{Al}}^{\mathrm{s}}$
Al–0.7Ni	0.32
Al–0.6Ni	0.46
Al–0.5Ni	0.56
Al–0.4Ni	0.68
Al–0.3Ni	0.82
Al–0.2Ni	0.87
Al–0.1Ni	0.96
Al	1

### Cluster identification

**Figure 5:**
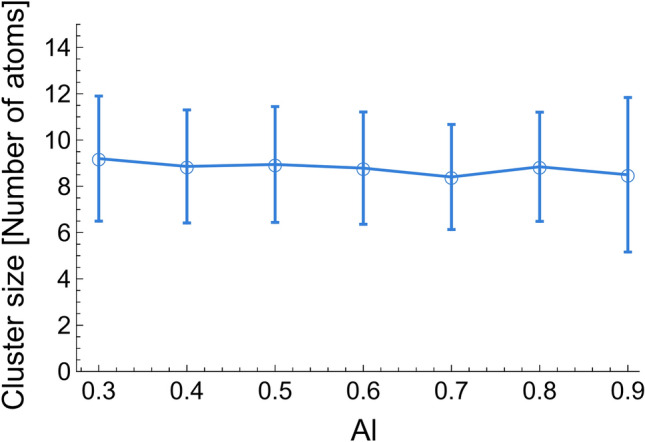
Cluster size in Al–Ni alloys with different Al content obtained from MD simulations.

**Figure 6:**
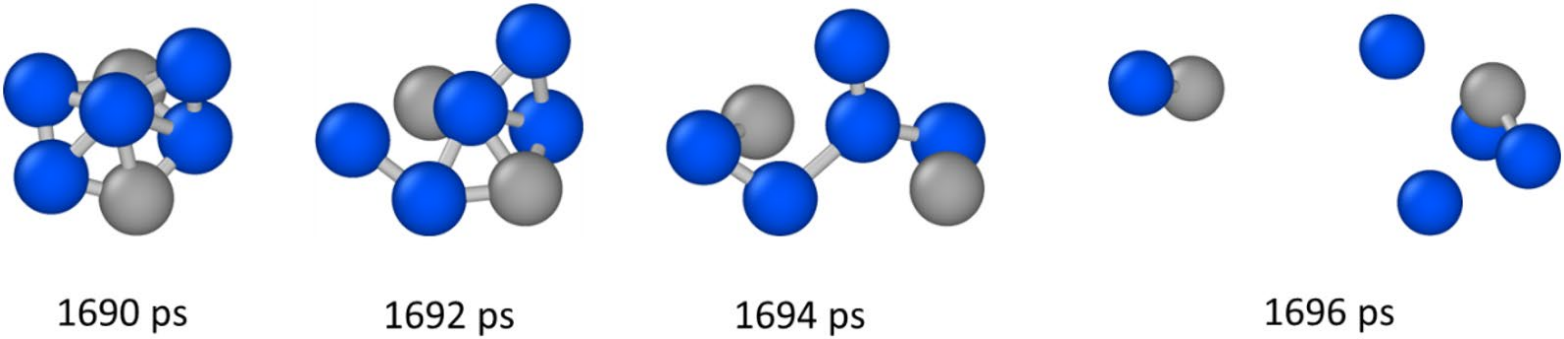
Dissociation of a cluster in Al–0.5Ni. Al atoms in gray. Ni atoms in blue.

Cluster identification provided interesting results to unravel the relationship between composition and surface tension of Al–Ni alloys. It was observed that clusters constantly formed and dissociated, with life from 2 to 10 ps, and sizes for all alloys ranged from 6 to 12 atoms, as shown in Fig. 5. An example of a cluster forming and dissociating in Al–0.5Ni is shown in Fig. 6.

### Influence of composition on surface tension

**Figure 7:**
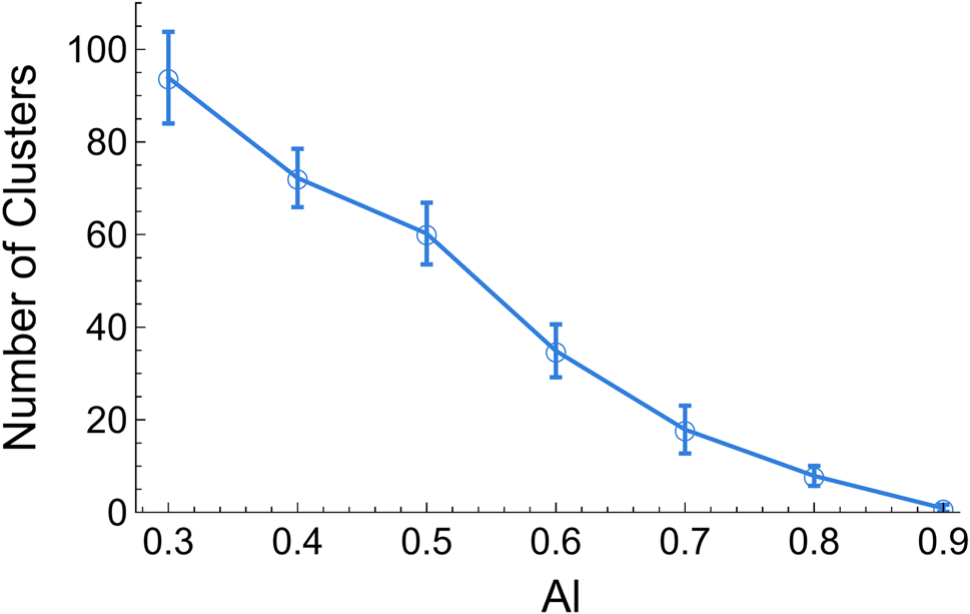
Number of clusters in Al–Ni alloys as obtained from MD simulations for different Al content (The error bars in this figure represent the standard deviation).

**Figure 8:**
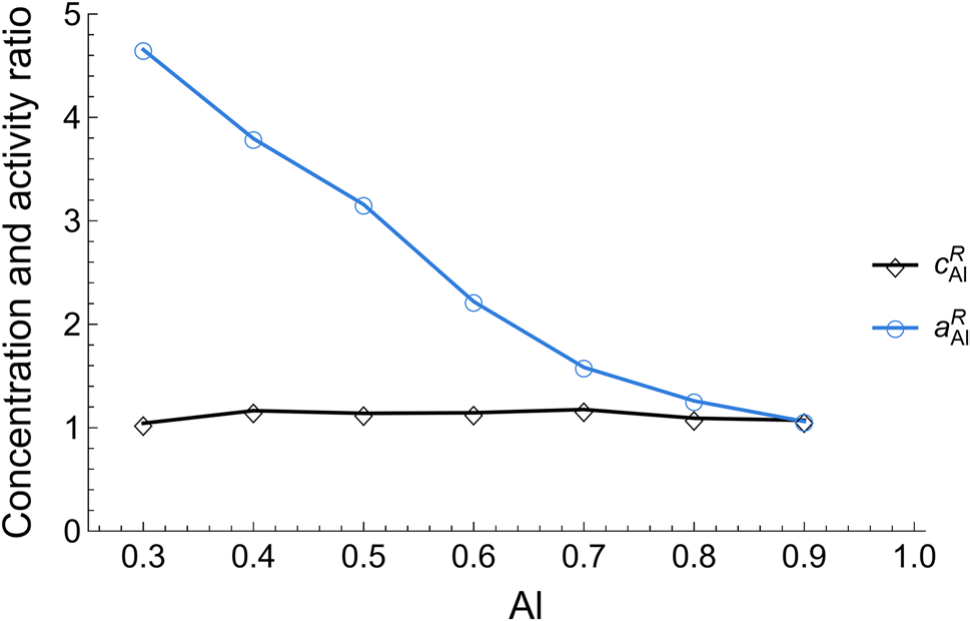
Activity and concentration ratios of Al–Ni alloys.

As mentioned before, one of the characteristics of real solutions is the possible formation of atomic clusters with short-range order. As observed from Fig. 7, there is an obvious general trend between composition and the number of clusters: the number of clusters decreases linearly as the Al content increases. Quite interestingly, the number of Al–Ni clusters is not the same for all Al–Ni alloy compositions, and, consequently, one could suspect that not all the alloys have the same degree of deviation from the ideal solution. This can be verified by comparing $a_{Al}^{R}$ with $c_{Al}^{R}$, since their difference is proportional to the magnitude of deviation from an ideal solution (Fig. 8). It should be mentioned that $a_{Al}^{R}$ was calculated using the Butler’s equation (Eq. 10).

Figure 8 shows that Al–0.2Ni and Al–0.1Ni behavior are close to the like ideal solutions. As such, the activity ratio is approximately equal to its concentration ratio. They were expected to have no or few Al–Ni clusters, consistent with the result shown in Fig. 7. Contrastingly, Al–0.7Ni, Al–0.6Ni, Al–0.5Ni, Al–0.4Ni, and Al–0.3Ni showed varying amounts of deviation from the ideal solution. Moreover, the amount of deviation followed the same trend as the number of clusters, i.e., a larger deviation from the ideal state corresponded with a higher number of clusters.

**Figure 9:**
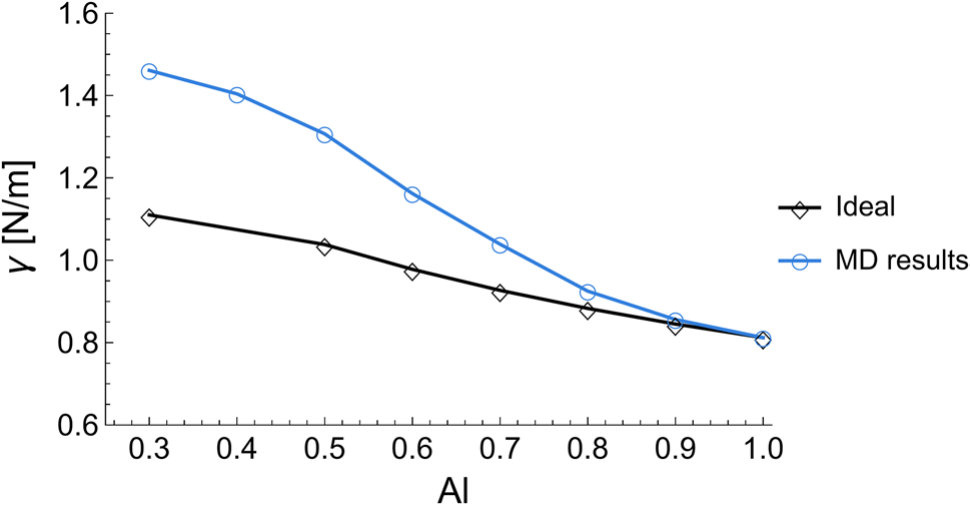
Surface tension of Al–Ni alloys as obtained using MD and using the ideal solution model.

The effect of clusters on Al–Ni surface tension was studied by comparing the surface tension obtained with MD with an equivalent ideal one using the Guggenheim equation (Eq. 3), as seen in Fig. 9. It was observed that by increasing the number of clusters, the magnitude of deviation from an ideal solution increases (Fig. 7 and 9). The presence of clusters decreased Al surface content, i.e., decreased Al surface segregation. This corroborated the observation by Novakovic et al.^25^ that clusters increase surface tension. However, in contrast with these authors, the MD clusters were actually characterized and not merely obtained from a thermodynamic model assumed a priori to be correct.

**Figure 10:**
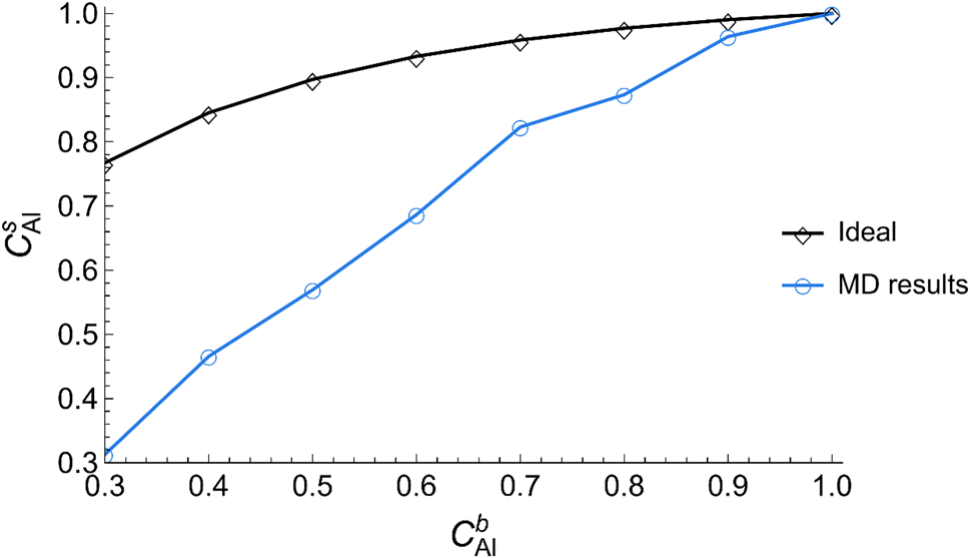
Surface composition of Al–Ni alloys obtained using MD and using the ideal solution model.

As an attempt to understand how clusters influence the surface tension, the surface obtained with MD and an equivalent ideal solution using Eq. (2) were compared in Fig. 10, where the presence of clusters decreased the Al surface content, i.e., decreased Al segregation. This corroborated once again the observation by Novakovic et al.^25^.

The influence of surface Al content on surface tension can be understood as follows: In any A–B binary alloy, one element will always have lower surface tension and will attempt to segregate onto the surface to lower the energy of the system. If, somehow, the segregation of the element with lower surface tension is hindered, then, naturally, the surface tension of the system increases. In an Al–Ni system, Al is the element with the lowest surface tension and thus will attempt to segregate to the surface^24^. The ideal solution model shows the unhindered Al segregation (black curve in Fig. 10) and its corresponding surface tension (black curve in Fig. 9). However, in the Al–Ni system, at compositions with < 70 at.% Al content, Al segregation will be hindered by clusters, thus reducing the surface Al content (blue curve in Fig. 10) and consequently increasing the surface tension (blue curve in Fig. 9). Therefore, it was suggested that clusters increase surface tension by decreasing Al surface segregation.

**Figure 11:**
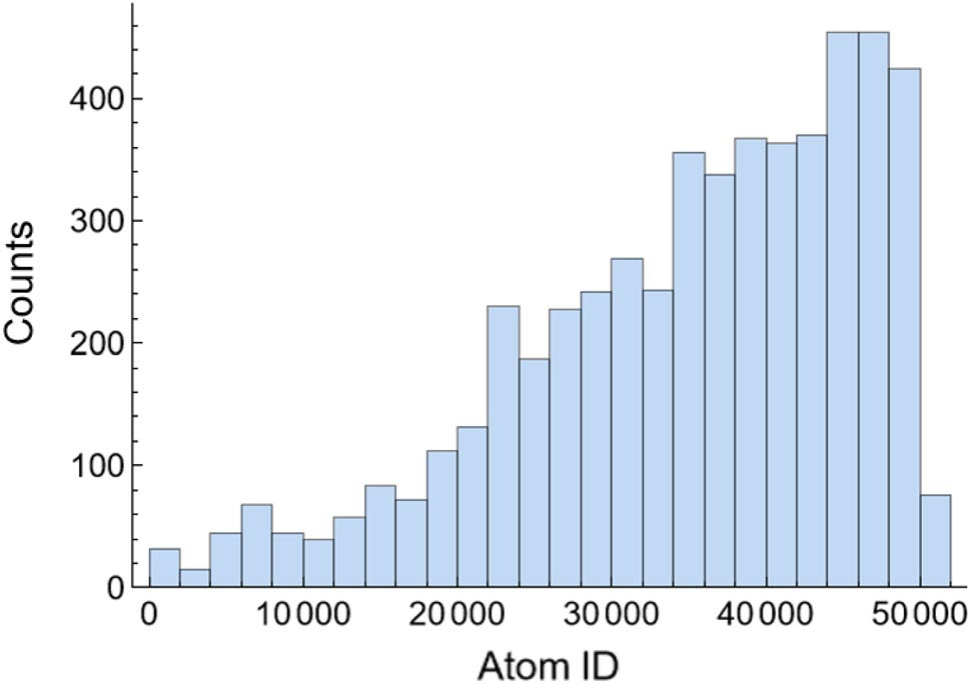
Histogram of Al atoms on the Al–0.5Ni surface.

Once it was proposed that clusters alter surface tension by hindering Al surface segregation, the remaining question was, why do clusters affect surface segregation? The suggested answer to this question was based on two observations: the Al atoms do not remain at the surface, and the Al atoms trapped in clusters travel less than their free counterparts (the term trapped atoms makes reference to atoms entrapped by a cluster while the term free atoms denotes atoms not trapped in clusters). These two observations, and their role in explaining how clusters modify the surface composition, are addressed using Al–0.5Ni as an example. To study whether the atoms remained at the surface, a histogram with the number of times that a specific atom appeared on the surface during the last 10,000 time steps is shown in Fig. 11. As can be seen from this figure, the specific atoms on the surface change with time. This is caused by the continuous motion of atoms across the slabs.

**Figure 12:**
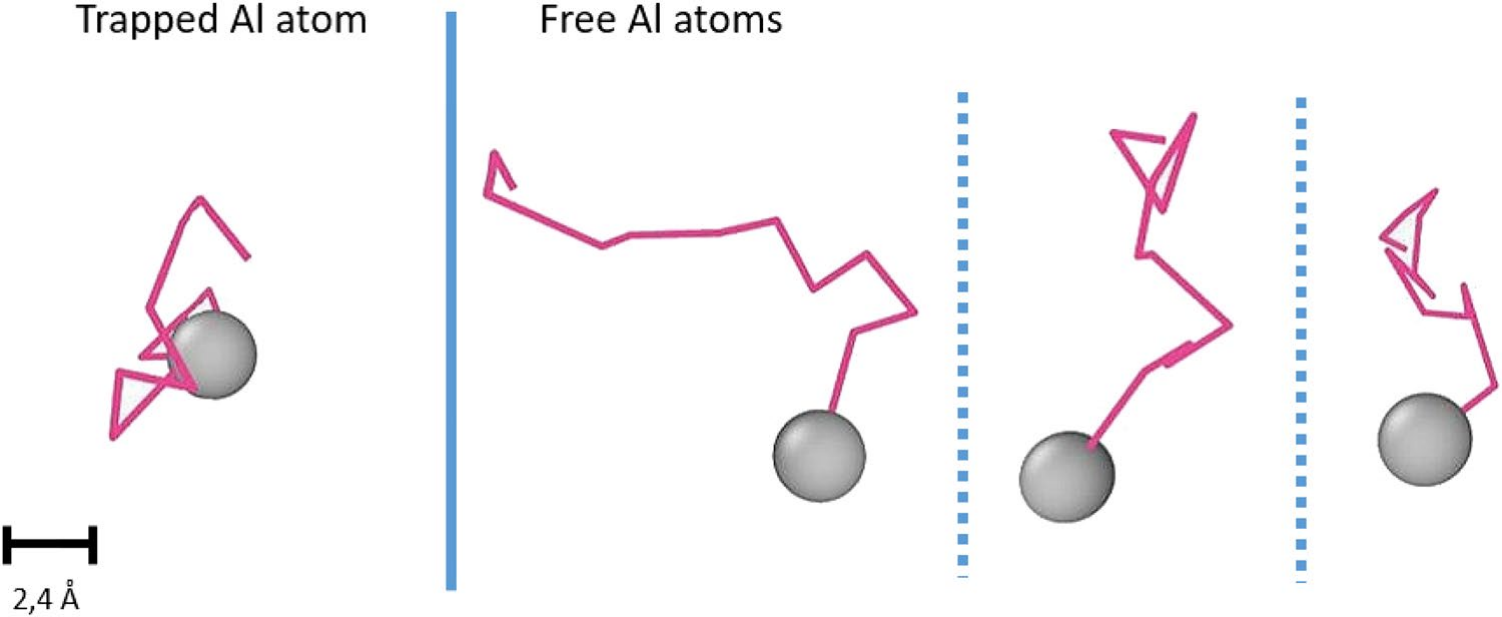
Trajectory of trapped and free Al atoms in Al–0.5Ni alloy.

The characteristics of the movement, however, were not the same for all atoms. Figure 12 compares the trajectory during the last 10,000 time steps of one of the trapped Al atoms with the trajectory of three random free Al atoms in the same time interval. It can be seen that trapped Al atom traveled less distance than their free counterparts. This observation was generalized to the whole system by comparing the mean square travel distance (MSTD) of the free and trapped Al atoms during the same last 400,000 time steps. This value of both groups was given by Eqs. (12) and (13), respectively, where (*t*) is the position of the atom at time step *t*, and (*t*_0_) is the reference position. It should be reminded that the clusters were forming and dissociating, thus the Al atoms’ status as free or trapped atoms varied during the studied time length. Therefore, it was decided to use the position at the previous time step as the reference position to avoid confusing free and trapped behavior.

12 $$MSTD^{trapped} = \frac{1}{{N^{trapped} }}\mathop \sum \limits_{j}^{{N^{trapped} }} \left| {x_{j}^{trapped} \left( t \right) - x_{j}^{trapped} \left( {t_{0} } \right)} \right|^{2}$$

13 $$MSTD^{free} = \frac{1}{{N^{free} }}\mathop \sum \limits_{j}^{{N^{free} }} \left| {x_{j}^{free} \left( t \right) - x_{j}^{free} \left( {t_{0} } \right)} \right|^{2}$$

**Figure 13:**
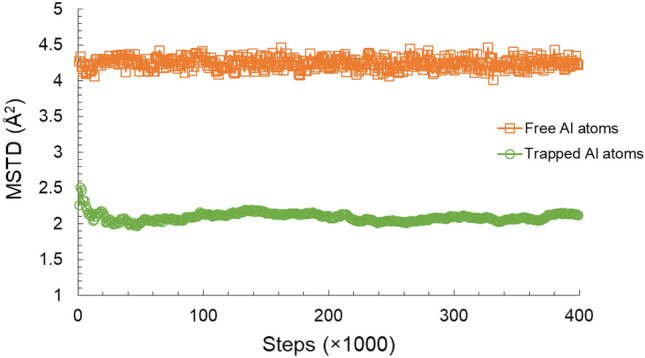
MSTD of free and trapped Al atoms at Al–0.5Ni alloy.

As can be seen from Fig. 13 trapped Al atoms traveled less than their free counterparts. Notice the MSTD shown in this figure does not increase with time because the reference position was the previous atom position.

## Conclusion

Binary Al–Ni alloys have been, and continue to be, the focus of extensive research. In particular, the surface tension of liquid Al–Ni alloys is of quite an interest since this property influences the final microstructure as well as the presence of defects. Despite this interest, the relationship between composition and surface tension of liquid Al–Ni alloys is not yet fully understood. This is due to the fact that, at some compositions, Al and Ni strongly interact, forming Al–Ni atomic clusters with short-range order. Then, the thermodynamic models normally applied to many alloys to predict surface tension do not work so well. This difficulty is further compounded by experimental complications and limitations, which cause that some key properties useful to understand the Al–Ni surface tension behavior can only come from thermodynamic models.

This work used Molecular Dynamics in an effort to understand the complex relationship between composition and surface tension of liquid Al–Ni alloys. This simulating technique has the great advantage that it allows for direct prediction of atomistic behavior.

By comparing the results from the simulations with those of an equivalent ideal solution, it was possible to infer the role of Al–Ni clusters playing in surface tension. It was found clusters appear in alloys that show a large deviation from the ideal solution, a larger deviation from the ideal state corresponded with a higher number of clusters.

It was proposed that these Al–Ni clusters increase surface tension by decreasing the surface Al content. They achieve this by entrapping Al atoms and hindering their travel, which in turn decreases their probability of reaching the surface. Indeed, there is an observable relationship between the relative amount of Al atoms trapped in clusters and the difference in Al content between the surface alloy and its corresponding ideal alloy. This entrapment of Al atoms leads to an increase in surface tension because this property is related to the amount of Al at the surface. Since Al has a lower surface tension than Ni, it tends to segregate onto the surface to lower the overall energy of the system. Therefore, fewer Al atoms at the surface conduces to an increase in surface tension compared to the ideal solution. As not all Al–Ni alloys present the same amount of deviation from the ideal state, the influence of clusters on surface tension will not be the same. This leads to the non-monotonous, non-concave curve that is characteristic of this system.

In summary, although surface tension only depends on what happens at the surface, it may seem that in Al–Ni alloys, the bulk indirectly plays a role in surface tension. This is because the bulk composition will influence cluster formation, which will modify the surface composition, which in turn, will modify surface tension.

In the future, this method could be applied to other binary systems in order to investigate their behavior and compare it with ideal, regular, and real solutions.

## Supplementary Information

Supplementary Information.

## Data Availability

The datasets generated and/or analysed during the current study are not publicly available as the data also forms part of an ongoing study but are available from the corresponding author on reasonable request.
