# Author Correction: Modeling of surface phenomena of liquid Al–Ni alloys using molecular dynamics

**DOI:** 10.1038/s41598-023-39129-y

**Published:** 2023-07-25

**Authors:** Hadassa Juárez, Ensieh Yousefi, Anil Kunwar, Youqing Sun, Muxing Guo, Nele Moelans, David Seveno

**Affiliations:** 1 Department of Materials Engineering, KU Leuven, Kasteelpark Arenberg 44, Box 2450, 3001 Leuven, Belgium; 2 Faculty of Mechanical Engineering, Silesian University of Technology, Konarskiego 18A, 44‑100 Gliwice, Poland

Correction to: *Scientific Reports* 10.1038/s41598-023-31844-w, published online 21 March 2023

The original version of this Article contained errors in References 26 and 29, which were incorrectly given as:

26. Yousefi, E. *et al.* Surface tension of aluminum-oxygen system: a molecular dynamics study. *Acta Mater.* **221**, 9–11 (2021).

29. Kunwar, A. *et al.* Multi-phase field simulation of Al_3_Ni_2_ intermetallic growth at liquid Al/solid Ni interface using MD computed interfacial energies. *Int. J. Mech. Sci.*, **215**(5), 2021, pp. 0–8 (2022).

The correct references are listed below:

26. Yousefi, E. *et al*. Surface tension of aluminum-oxygen system: a molecular dynamics study. *Acta Mater*. **221**, 117430 (2021).

29. Kunwar, A. *et al*. Multi-phase field simulation of Al_3_Ni_2_ intermetallic growth at liquid Al/solid Ni interface using MD computed interfacial energies. *Int. J. Mech. Sci.*, **215**, 106930 (2022).

The original Article has been corrected.

