# Supplementary material for: Modeling of surface phenomena of liquid Al–Ni alloys using molecular dynamics
Source: Sci Rep. 2023 Mar 21;13:4642. doi: 10.1038/s41598-023-31844-w (PMC10030778; doi:10.1038/s41598-023-31844-w)
Supplement: Supplementary file 1 — Supplementary Information. [file 41598_2023_31844_MOESM1_ESM.pdf]

Supplementary material for

Modeling of Surface Phenomena of Liquid Al-Ni Alloys Using Molecular Dynamics

Hadassa Juárez[a, †], Ensieh Yousefi[a,*, †], Anil Kunwar[b,a], Youqing Sun[a], Muxing Guo[a], Nele Moelans[a], David Seveno[a]

[a] *Department of Materials Engineering, KU Leuven, Kasteelpark Arenberg 44, Leuven 3001 , Belgium*

[b] *Faculty of Mechanical Engineering, Silesian University of Technology, Konarskiego 18A, 44-100 Gliwice, Poland*

* Corresponding author phone: +32 16 37 64 29, email: ensieh.yousefi@kuleuven.be

This file includes:

Mean molar surface area

Guggenheim model

Quasi-Chemical Approximation (QCA)

Force field: Surface tension of aluminum was measured by using different Al-Ni force fields to check their accuracy.

Simulation setup: In this section, the effect of initial configuration and size of the system on surface tension value were studied.

Figure A.4: This figure shows the total energy (potential energy + kinetic energy) of an Al-0.5Ni system through time to show the system is at an equilibrium state during data collecting.

Figure A.5: This figure shows the evolution of surface tension as a function of time. It also indicates stable surface tension was achieved after 0.6 ns.

[†]These authors contributed equally to this paper

Mean molar surface area

Mean molar surface (α) area is measured as follows considering the mean molar surface area of each pure component *i* (S_i):

$$\alpha = \sum_i c_i^b S_i \qquad \textit{Equation 1}$$

S_i could be measured from the atomic mass, M_i and the density, ρ_i [6]:

$$S_i = 10.91 N_A \left(\frac{M_i}{\rho_i}\right)^{2/3} \qquad \textit{Equation 2}$$

Assume that the atoms of component i are spherical with radius r_i. Then the molar surface area (S_i) is obtained as:

Equation 3

$$S_i = \pi . r_i^2 . \frac{N_A}{f_i}$$

which N_A is Avagadro number, f_i is the surface packing fraction of pure component *i* (dimensionless).

The molar volume of component *i* (V_i) is obtained as:

Equation 4

$$V_i = \frac{4}{3} . \pi . r_i^3 . \frac{N_A}{f_b}$$

f_b is the bulk packing fraction of pure component i (dimensionless).

By combining equations (1) and (2):

Equation 5

$$S_i = L . V_i^{2/3} . N_A^{1/3}$$

Equation 6

$$L = \frac{\pi}{f_i} . \left(\frac{3 . f_b}{4\pi}\right)^{2/3}$$

Based on [1], the value of L is set to be 1.091 for liquid metals which is used in this study.

Guggenheim model

An approach to obtain the surface tension of an ideal alloy was proposed by Guggenheim [2]. This model, called the Guggenheim equation, is based on the ratio between the molecular partition functions of the surface (f_i^s) and that of the bulk f_i^b of each component. For an ideal solution, this ratio can be expressed as:

$$\frac{f_i^b}{f_i^s} = Exp\left[\frac{\gamma_i \alpha}{k_B T}\right] \qquad \text{Equation 7}$$

where γ_i is the surface tension of the pure component, k_B is the Boltzmann constant, T is the temperature (K), α is mean molar surface area. $\frac{f_i^b}{f_i^s}$ ratio can be used in a simple rule of mixtures to obtain the surface tension of an alloy (γ):

$$Exp\left[\frac{-\gamma\alpha}{k_B T}\right] = \sum_i c_i^b \left[\frac{f_i^b}{f_i^s}\right] \qquad \text{Equation 8}$$

By combining Equation 7 and 6, the Guggenheim equation is obtained as:

$$Exp\left[\frac{-\gamma\alpha}{k_B T}\right] = \sum_i c_i^b \left[\frac{-\gamma_i \alpha}{k_B T}\right] \qquad \text{Equation 9}$$

Quasi-Chemical Approximation (QCA)

For QCA, it is proposed to obtain the activities from the Grand Partition Function of the Bulk, Ξ^b, and the Grand Partition Function of the Surface, Ξ^s. In Equation 10 and Equation 11, E is the configurational energy of a specific alloy, N_A and N_B are the number of atoms of A and B, q_A^N and q_B^N are the atomic partition functions of A and B, and μ_A and μ_B are their respective chemical potentials.

$$\Xi^b = \sum_{E^b} q_A^{N^b}(T)\, q_B^{N^b}(T) Exp\left[\frac{\mu_A^b\, N_A^b + \mu_A^b\, N_A^b - E^b}{k_b T}\right] \qquad \textit{Equation 10}$$

$$\Xi^{s} = \sum_{E^{s}} q_{A}^{N^{s}}(T)\, q_{B}^{N^{s}}(T) Exp\left[\frac{\mu_{A}^{s}\, N_{A}^{s} + \mu_{A}^{s}\, N_{A}^{s} - E^{s}}{k_{b}T}\right] \qquad \textit{Equation 11}$$

Additionally, surface coordination fractions are required because, although the surface and the bulk are considered to have the same type of packing, the surface monolayer has reduced coordination in comparison with the bulk [3].

$$p + 2q = 1 \qquad \textit{Equation 12}$$

Considering any atom in the surface layer, the fraction number of its neighbors in the surface layer is p, and the fraction number of its neighbors in the next layer through the bulk is q.

By using the Grand Partition Functions (Equation 13 and Equation 14), the activities for this model are:

$$a_{A} = \left(\frac{\beta - 1 + 2\, c_{A}}{c_{A}(1 + \beta)}\right)^{Z/2} \qquad \textit{Equation 13}$$

$$a_{B} = \left(\frac{\beta + 1 - 2\, c_{B}}{c_{B}(1 + \beta)}\right)^{Z/2} \qquad \textit{Equation 14}$$

In Equation 13 and Equation 14, Z is the coordination number and β is an auxiliar variable defined by Equation 15, where Ω is the interaction energy. This last parameter involves the difference between the A-B bond energy and the average of A-A and A-B bond energies (Equation 16) [4].

$$\beta = \left(4\, c_{i}(1 - c_{i}) Exp\left[\frac{2\,\Omega}{Z\, k_{b}\, T}\right] + (1 - 2\, c_{i})^{2}\right)^{1/2} \qquad \textit{Equation 15}$$

$$\Omega = Z\left(\varepsilon_{AB} - \frac{\varepsilon_{AA} + \varepsilon_{BB}}{2}\right) \qquad \textit{Equation 16}$$

Thus, by combining Equation 1 (in the text of the paper), Equation 12, Equation 13, and Equation 14, the relationship between surface tension and composition of a regular alloy is given by the QCA model as:

$$\gamma = \gamma_i + \frac{N_A\, k_B\, T}{\alpha} \ln\left[\frac{c_i^s}{c_i^b}\right] + \frac{N_A\, k_B T}{\alpha}\left(p \ln\left[\frac{a_i^s}{a_i^b}\right] - q\; ln\left[a_i^b\right]\right) \qquad \textit{Equation 17}$$

In most literature, Equation 17 is usually expressed by β instead of using a_i^s and a_i^b by helping Equation 17 which, after some simplification, finally leads to Equation 18:

$$\gamma = \gamma_i + \frac{N_A\, k_B\, T(2 - p\, Z)}{2\,\alpha} \ln\left[\frac{c_i^s}{c_i^b}\right] + \frac{N_A\, k_B T\, Z}{2\,\alpha}\left(p \ln\left[\frac{(\beta^s - 1 + 2\, c_A^s)(1 + \beta^b)}{(\beta^b - 1 + 2\, c_A^b)(1 + \beta^s)}\right] - q \ln\left[\frac{(\beta^b - 1 + 2\, c_A^b}{c_A^b(1 + \beta^b)}\right]\right) \qquad \textit{Equation 18}$$

Using the CFM model, a_i^s can be expressed as:

$$\ln a_i^s = p \ln\left[a_i^b(c_i^s)\right] + q \ln\left[a_i^b(c_i^b)\right] \qquad \textit{Equation 19}$$

Force field:

7 different force fields developed to describe Aluminum-Nickel (or more) material are listed in Table A.1 (Embedded Atom Method alloy (EAM/alloy), Embedded Atom Method and charge transfer ionic (EAM+CTI) Reactive force field (ReaxFF), and charge optimized many-body (COMB)). To compare

their accuracy in predicting surface tension, the surface tension of aluminum (mN/m) at 1200 K was measured and compared to experimental results considered a reference value. Tables A.2 is sorted out from the best to the worst force field for surface tension. Based on table A.2, the Zhou force field is the best potential regarding the prediction of surface tension.

Table A.1 Aluminum-Nickel force fields

Force field	Developer of force field	Year
**EAM/alloy**	Baskes [5]<br>Al-Ni-H	1995
	Mishin1 [6]<br>Al-Ni	2002
	Mishin2 [7]<br>Al-Ni	2004
	Mishin3 [8]<br>Al-Ni	2009
**EAM+CTI**	Zhou [9]<br>Al-Ni-O	2004
**Reaxff**	Van Duin [10,11]	2015
**COMB**	Kumar [12]	2015

Table A.2 Surface tension of Aluminum

Force field	Surface tension (mN/m)	|Error| (%)<br>Reference = 864 mN/m
**Zhou**	882.7	2.4
**Mishin 1**	765.50	11.4
**Mishin 3**	721.13	16.5
**Baskes**	1070.00	23.8
**Mishin2**	623.49	27.9
**COMB**	580.54	32.9
**Reaxff**	184.52	78.7

Simulation setup

Initial configuration

For the initial configuration, three different types were tested: a center Al slab sandwiched between two Ni slabs (Type A), a center Ni slab sandwiched between two Al slabs (Type B), and a random configuration, as discussed in Chapter IV (Type C). These three types are illustrated in Figure A.1. All initial configurations yielded similar surface tension results, with a variation of <2% (Figure A.2).

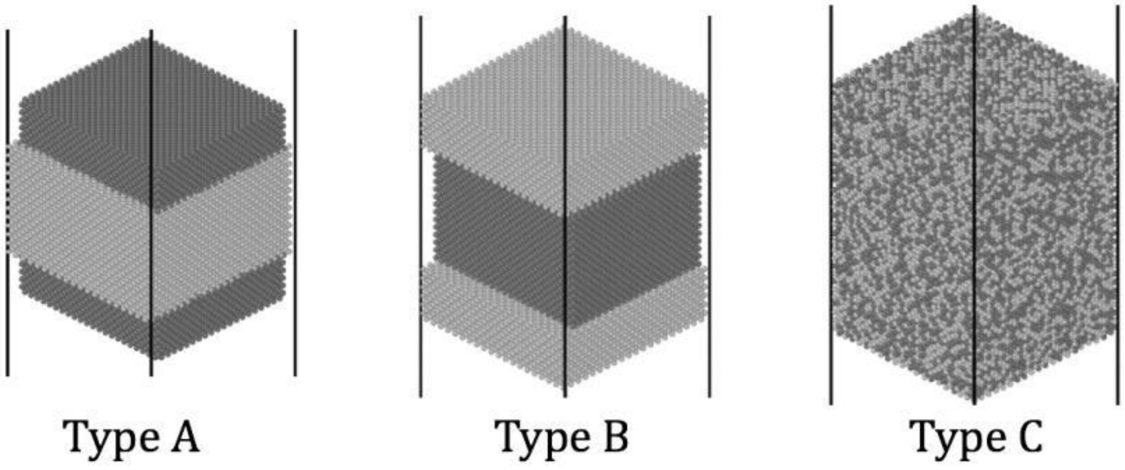

Figure A.1 Tested initial configurations. Al atoms in light gray. Ni atoms in dark gray

System size

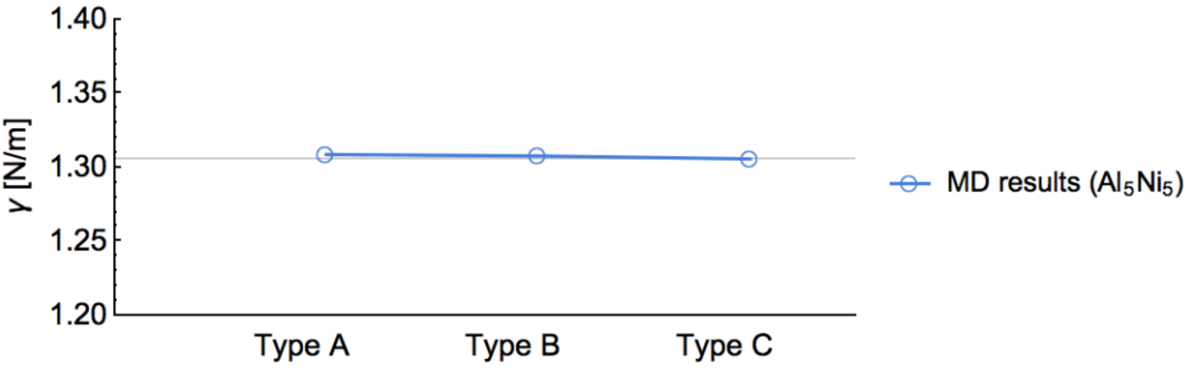

Figure A.2 Surface tension of Al5Ni5 for different initial configurations

The influence of system size was explored for three different number of atoms: 20,000, 50,400, and 60,000. As reflected from Figure A.3, the surface tension did not significantly change, presenting a

variation of <2%. Thus, the tested system sizes were above the limit where they significantly influence surface tension.

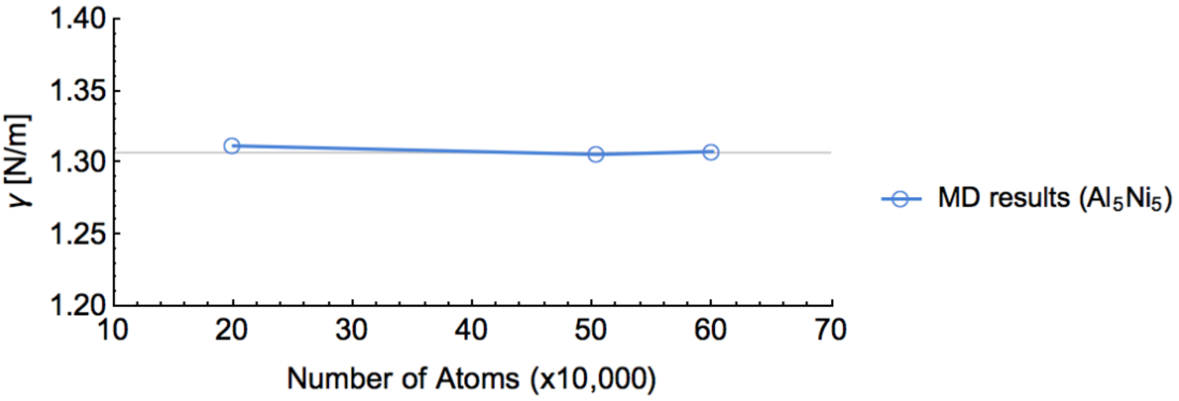

Figure A.3 Surface tension of Al-0.5Ni for different system size

Total energy of Al-0.5Ni system:

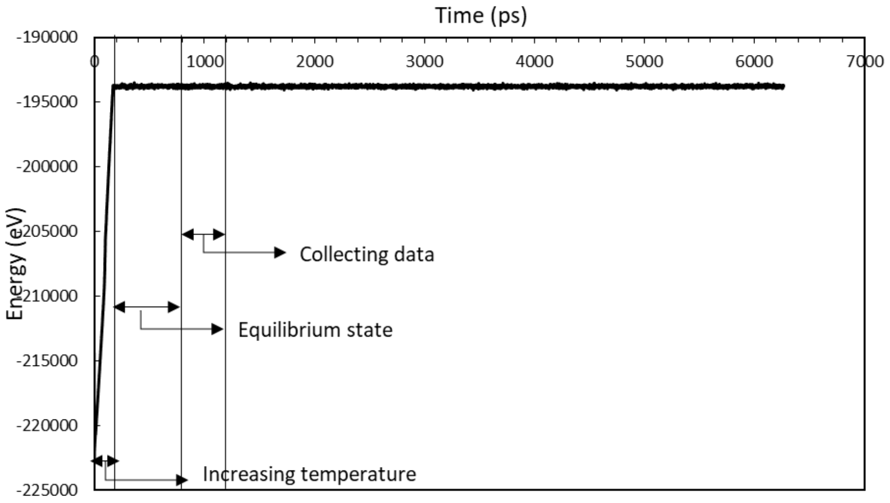

Figure A.4 Total energy of Al-0.5Ni System versus time.

Calculated surface tension using the mechanical approach as a function of time:

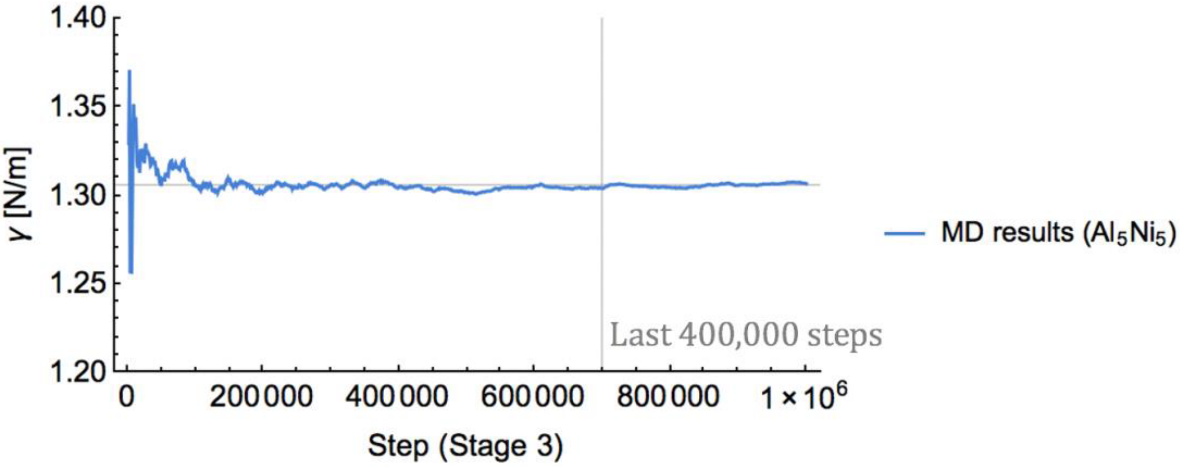

Figure A.5 Evolution of surface tension with number of steps

Reference

[1] T. Tanaka and T. Iida. Application of a thermodynamic database to the calculation of surface tension for Iron-Base Liquid Alloys. 1994;Vx(1):21–8,

[2] E.A. Guggenheim. Statistical Thermodynamics of the Surfce of a Regular Solution. Transactions of the Faraday Society. 1945;41:150–6,

[3] R. Novakovic and D. Zivkovic. Thermodynamics and surface properties of liquid Ga-X (X = Sn, Zn) alloys. Journal of Materials Science. 2005;40(9–10):2251–7,

[4] Porter, David. Phase Transformations in Metals and Alloys. 3rd ed. Boca Raton, FL.: CRC Press; 2009.

[5] M.I. Baskes, X. Sha, J.E. Angelo, and N.R. Moody. Trapping of hydrogen to lattice defects in nickel. Modelling and Simulation in Materials Science and Engineering. 1997;5(6):651–2,

[6] Y. Mishin, M.J. Mehl, and D.A. Papaconstantopoulos. Embedded-atom potential for B2-NiAl. Physical Review B - Condensed Matter and Materials Physics. 2002;65(22):1–14,

[7] Y. Mishin. Atomistic modeling of the γ and γ′-phases of the Ni-Al system. Acta Materialia. 2004;52(6):1451–67,

[8] G.P. Purja Pun and Y. Mishin. Development of an interatomic potential for the Ni-Al system. Philosophical Magazine. 2009;89(34–36):3245–67,

[9] X.W. Zhou, H.N.G. Wadley, J.S. Filhol, and M.N. Neurock. Modified charge transfer-embedded atom method potential for metal/metal oxide systems. Physical Review B - Condensed Matter and Materials Physics. 2004;69(3)

[10] K. Chenoweth, A.C.T. Van Duin, and W.A. Goddard. ReaxFF reactive force field for molecular dynamics simulations of hydrocarbon oxidation. Journal of Physical Chemistry A. 2008;112(5):1040–53,

[11] Y.K. Shin, H. Kwak, C. Zou, A. V Vasenkov, and A.C.T. Van Duin. Development and

Validation of a ReaxFF Reactive Force Field for Fe / Al / Ni Alloys : Molecular Dynamics Study of Elastic Constants , Di ff usion , and Segregation. 2012;

[12] A. Kumar et al. Corrigendum: Charge optimized many-body (COMB) potential for dynamical simulation of Ni–Al phases. Journal of Physics: Condensed Matter. 2015 Dec 2;27(47):336302,
